# Comparison of bacterial communities between midgut and midgut contents in two silkworms, *Antheraea pernyi* and *Bombyx mori*

**DOI:** 10.1038/s41598-020-69906-y

**Published:** 2020-07-31

**Authors:** Huan Wang, Jing-Yu Zhang, Xiao-Meng Wang, Hua-Lei Hu, Run-Xi Xia, Qun Li, Xu-Wei Zhu, Tian-Mao Wang, Yan-Qun Liu, Li Qin

**Affiliations:** 1 Department of Sericulture, College of Bioscience and Biotechnology, Shenyang Agricultural University, 120 Dongling Road, Shenyang, 110866 China; 2 Henan Sericultural Research Institute, Zhengzhou, 450008 China; 3 Heilongjiang Sericultural Research Institute, Ha’erbin, 150086 China; 4 Present Address: Gan & Lee Pharmaceuticals, Beijing, 101109 China

**Keywords:** Microbiome, Symbiosis

## Abstract

Bacterial communities living inside the midgut of insects have been attracting increasing interest. Previous studies have shown that both the midgut and midgut contents harbor bacterial communities. However, whether the bacterial communities of the insect midgut are similar to those of the insect midgut contents (including the peritrophic membrane, food particles, and digestive fluids secreted by the midgut in this study) remains unknown. In the present study, we analyzed two economically important silkworms, the Chinese oak silkworm *Antheraea pernyi* (Lepidoptera: Saturniidae) and the mulberry silkworm *Bombyx mori* (Lepidoptera: Bombycidae), through Illumina MiSeq technology to address this issue. In *A. pernyi* larvae, 17 phyla and 162 genera were found in the midgut, while 7 phyla and 36 genera were found in the midgut contents. For *B. mori* larvae, 30 phyla and 465 genera were found in the midgut, but 22 phyla and 344 genera were found in the midgut contents. This evidence from the two silkworms suggests that the bacterial composition and diversity in the midgut are more diverse than those in the midgut contents. Principal component analysis revealed a significant difference in the bacterial community structure between the midgut and midgut contents of *B. mori*. To our knowledge, this is the first study to compare the bacterial communities between the midgut and midgut contents in insects, and the results will provide useful information for probing the functional differentiation within the midgut in the future.

## Introduction

In insects, the midgut is composed of a single layer of epithelial cells and serves as the main place where food is digested and absorbed. There are various kinds of microbiota residing in the insect midgut, including both nonpathogenic and pathogenic bacteria consumed with food^1^. It is increasingly recognized that these bacteria play many important roles in their hosts, including decomposing toxins and insecticides, defending against parasites and other pathogens, transferring signals, improving immunity and behavior, and producing nutrients to supplement poor diet and digestion^2–4^. With recent advances in next-generation sequencing (NGS), the bacterial communities associated with insects have attracted increasing interest, mainly because of their ecological and economic importance^5–7^. Previous studies have demonstrated that the composition and structure of the intestinal bacterial community are dynamic and can vary with lifestyle, type of feed, living conditions, insect species, strain and sex^6–9^. Thus, the characterization of the gut bacterial community is essential for a comprehensive understanding of the biology and ecology of the insect species of interest.

The peritrophic membrane (PM) is a special semipermeable membrane structure in the digestive tract of insects that is located between food and midgut epithelial cells. The PM is thought to protect midgut epithelial cells from mechanical damage and invasion by pathogens and toxins and act as a semipermeable membrane separating the midgut into different compartments, thereby playing important roles in digestion and absorption of nutrient substances^10,11^. Structurally, the PM divides the intestine into two parts: the area surrounding the PM (midgut) and the area inside the PM (abrasive food bolus, including food particles and the digestive fluids secreted by the midgut). A large number of studies have characterized the microbes in the intestines of many insects, with diverse sample preparation methods: some applied the whole midgut (including the midgut, PM, food particles, and digestive fluids) as samples^5,9,12^, some applied only the midgut as samples^6,13,15^, while others used the midgut contents (including the PM, food particles, and digestive fluids) as materials^14,16,17^. These previous studies have shown that both the midgut and midgut contents harbor bacterial communities^6,13–17^. However, whether the midgut and gut contents possess similar bacterial communities remains an issue that needs to be addressed. It has been evident that the PM provides a structural basis for the functional differentiation of different compartments within the midgut^11^. Therefore, a comparison of the bacterial community between the midgut and midgut contents will provide useful information for probing the functional differentiation within the midgut in the future.

In the present study, we used two economically important silkworm species, the Chinese oak silkworm *Antheraea pernyi* (Lepidoptera: Saturniidae) and the mulberry silkworm *Bombyx mori* (Lepidoptera: Bombycidae), to comprehensively understand the intestinal bacterial communities of silkworms. The Chinese oak silkworm *A. pernyi*, which is used for coarse silk production and as a human food source (larva, pupa and moth) in China, is commercially cultivated mainly in China, India, and Korea. Approximately 7 million kilograms of cocoons (pupae) are produced in China each year^18^. Presently, *A. pernyi* larvae are reared in the field on oaks (*Quercus* spp.)^20^. The mulberry silkworm *B. mori* plays important roles in silk fiber, animal protein, and bioreactor production and is an ideal model of Lepidoptera; unlike *A. pernyi* larvae, *B. mori* larvae are reared indoors using mulberry leaves (*Morus* spp.). Since nutrient absorption, nutrient utilization, and silkworm diseases are directly linked to the gut bacterial community of silkworm larvae^20,21^, information on the intestinal bacterial community will enhance our understanding of the diversity and function of silkworm intestinal microbiota.

A large body of information on the intestinal bacterial community of *B. mori* has been reported. For example, the gut bacterial community of *B. mori* has been shown to be impacted by the silkworm strain, forage, developmental stage, sex, and living condition linked to silkworms resistant to fluoride^9,13,14,17,22^. The health status of *B. mori* larvae has also been found to be linked with the gut bacterial community^14^^,^ and transient high temperature treatment can change the intestinal bacterial communities^17^. However, little is known about the intestinal bacterial communities of *A. pernyi*. Using the dilution culture method, Zou et al. (2011) isolated three bacterial genera, *Bacillus*, *Staphylococcus* and *Enterobacter*, from the midgut contents of larvae^23^^,^ and Huang et al. (2014) identified seven bacterial genera, *Bacillus*, *Staphylococcus*, *Enterobacter*, *Brevibacterium*, *Acinetobacter*, *Streptomyces*, and *Sporosarcina*, in the midgut of larvae^24^.

In this context, we undertook the present work to analyze the intestinal bacterial community of two important economical silkworms. Our primary objective was to determine whether the midgut shares similar bacterial communities with the midgut contents through Illumina MiSeq technology. The second objective of our study was to obtain information on the intestinal bacterial communities of *A. pernyi* larvae. Our experiments on *A. pernyi* and *B. mori* highlight the first evidence for a distinctive difference in the bacterial communities between the midgut and the midgut contents.

## Results

### Data collection description

**Figure 1:**
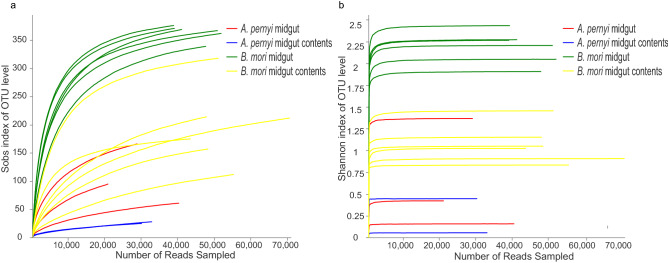
Richness rarefaction and Shannon index analysis of the silkworm samples used. (**a**) Rarefaction curves of OTUs clustered at 97% sequence identity across samples. (**b**) Rarefaction curves of the Shannon index according to OTU.

In the present study, we acquired four sets of data from 17 samples. Note that the bacterial communities of the midgut and the midgut contents were analyzed in parallel for each sample. We could not acquire a suitable PCR amplification product from one sample of *A. pernyi* midgut contents (*A. pernyi* midgut contents _1). After the low-quality reads were removed, the present study yielded a total of 30,039, 31,305, 45,984, and 53,309 valid reads per sample for *A. pernyi* midgut, *A. pernyi* midgut contents, *B. mori* midgut, and *B. mori* midgut contents, respectively, using Illumina MiSeq sequencing of 16S rRNA genes (Table S1). The average length of each read was 428–450 bp. More than 94.34% valid reads could be assigned to the genus level*.* Sequencing data were used to evaluate the diversity and richness of intestinal bacteria (Fig. 1). The richness rarefaction curves for individual samples began to plateau by the time all reads had been analyzed, indicating that the sequencing depth was sufficient to uncover most of the biodiversity in the larval intestine. The Shannon index was also used to estimate the bacterial diversity, and the Shannon rarefaction curves tended to plateau, indicating that the bacterial diversity varied in different samples.

### The intestinal bacterial communities in *A. pernyi* larvae

**Figure 2:**
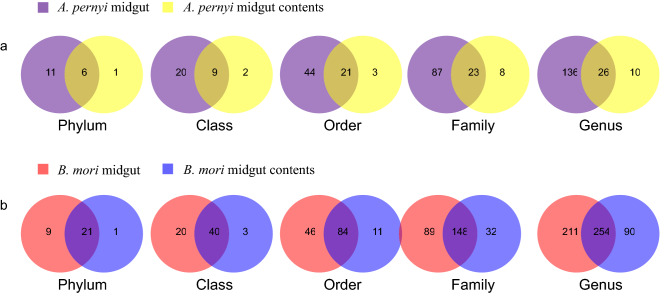
Shared bacterial types at different classification levels among samples. (**a**) Venn analysis of the midgut and midgut contents of *A. pernyi*. (**b**) Venn analysis of the midgut and midgut contents of *B. mori*.

In a preliminary experiment, we used *A. pernyi* larvae reared in the field in June 2017 to explore whether the midgut and midgut contents contained similar bacterial communities. In *A. pernyi*, the representative OTUs in the midgut were assigned to the different levels of taxonomical classification as follows (values for the midgut contents are shown in parentheses): 17 (7) phyla, 29 (11) classes, 65 (24) orders, 111 (31) families, 162 (36) genera and 206 (42) OTUs. Venn analysis showed that 6 phyla, 9 classes, 21 orders, 23 families, and 26 genera were common between the midgut and the midgut contents of *A. pernyi* larvae (Fig. 2a). These data indicated that the midgut possesses more diverse bacterial communities than the midgut contents.

**Figure 3:**
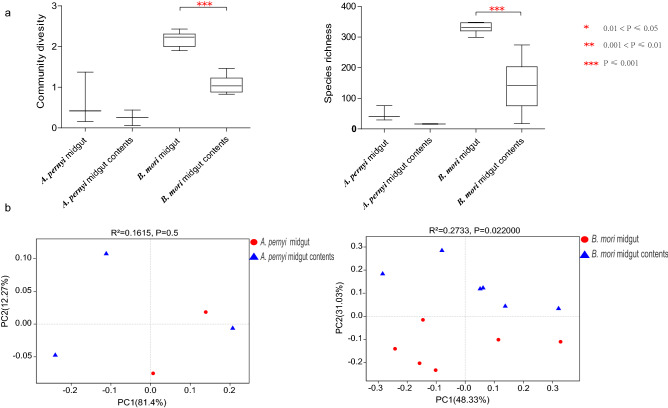
Bacterial community dynamics among samples. (**a**) Boxplot of species richness (number of OTUs) and community diversity measured by the Shannon index. Differences between the means of data of each treatment were compared by Student's *t* test. *** Indicates significant differences (P < 0.001). (**b**) PCoA plot showing variation in community structure between the midgut and the midgut contents. Variation in bacterial communities segregated strongly according to the midgut or midgut contents (ANOSIM test with 999 permutations, P < 0.05).

The estimated alpha-diversity of the intestinal bacteria in *A. pernyi* (Table 1) showed that the Chao index was higher in the midgut than in the midgut contents, but no certain trend was found for the Ace, Shannon, and Simpson indices between the midgut and the midgut contents. The boxplot of species richness (number of OTUs) and community diversity measured by the Shannon index indicated no significant difference between the midgut and midgut contents (Fig. 3a). The principal component ordination (PCoA) analysis did not cluster the two types of samples separately from one another.

**Table 1:** Comparison of diversity indices between the midgut and midgut contents.

Sample		Chao	Shannon	Ace	Simpson
*A. pernyi* midgut	_1	84.21	0.16	93.81	0.96
	_2	213.00	1.37	205.73	0.45
	_3	136.05	0.42	147.67	0.87
*A. pernyi* midgut contents	_2	38.20	0.45	69.28	0.78
	_3	62.00	0.056	167.39	0.99
*B. mori* midgut	_1	364.14	1.91	361.38	0.36
	_2	393.90	2.21	384.03	0.29
	_3	390.59	2.26	388.08	0.34
	_4	395.38	2.43	390.16	0.23
	_5	389.36	2.05	384.06	0.29
	_6	399.15	2.275	392.61	0.25
*B. mori* midgut contents	_1	191.87	1.05	194.74	0.45
	_2	169.65	0.83	229.51	0.54
	_3	263.39	0.91	271.11	0.58
	_4	342.85	1.467	336.06	0.39
	_5	196.08	1.02	186.29	0.66
	_6	254.32	1.15	268.67	0.43

**Figure 4:**
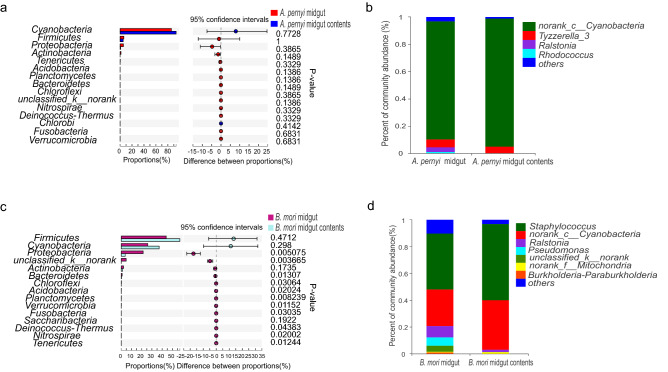
Comparison of the bacterial community between the midgut and midgut contents. (**a, c**) Wilcoxon rank-sum test bar plot at the phylum level between the *A. pernyi* midgut (n = 3) and midgut contents (n = 2) and between the *B. mori* midgut (n = 6) and midgut contents (n = 6). Differences were assessed by the Mann–Whitney U test. Significance was established at P < 0.05. (**b,d**) The dominant genus percentages for the midgut and midgut contents of *A. pernyi* and the midgut and midgut contents of *B. mori*, respectively. Bacteria with abundances less than 1% are classified as others.

For *A. pernyi* larvae, the dominant bacterial phyla in the midgut and the midgut contents were Cyanobacteria (85.90% and 93.82%), Firmicutes (6.25% and 5.20%), Proteobacteria (5.67% and 0.91%), Actinobacteria (1.58% and 0.06%), Tenericutes (0.34% and 0%), and Bacteroidetes (0.04% and 0.0015%) (Fig. 4a). The dominant bacterial genera in the midgut and the midgut contents were *norank_c__Cyanobacteria* (86.70% and 94.06%), *Tyzzerella_3* (5.86% and 4.86%), *Ralstonia* (3.42% and 0.04%), and *Rhodococcus* (1.13% and 0.04%) (Fig. 4b). Although the number of each taxon was different, the univariate analyses did not show any significant difference between sample types for this species. The reason for no significant difference between sample types for *A. pernyi* could be explained by the larger individual difference (see Discussion).

### Significant differences in bacterial communities between the midgut and midgut contents in *B. mori*

Here, another lepidopteran insect, *B. mori*, was used to verify the difference in bacterial communities between the midgut and the midgut contents found in *A. pernyi* larvae. In September 2017, we collected more samples from *B. mori* larvae that were reared indoors. In total, in *B. mori*, 30 (22) phyla, 60 (43) classes, 130 (95) orders, 237 (180) families, 465 (344) genera and 676 (481) OTUs were well represented in the midgut (values for the midgut contents area shown in parentheses). Venn analysis showed that 21 phyla, 40 classes, 84 orders, 148 families, and 254 genera were shared between the midgut and the midgut contents of *B. mori* larvae (Fig. 2b). The estimated alpha diversity of the intestinal bacteria showed that in *B. mori*, the Chao (*df* = 5, *t* = 6.24, P = 0.002), Ace (*df* = 5, *t* = 13.03, P = 0.000) and Shannon index (*df* = 5, *t* = 6.69, P = 0.002) was significantly higher in the midgut than in the midgut contents, and the Simpson index (*df* = 5, *t* = 5.50, P = 0.002) was lower in the midgut than in the midgut contents. These results also indicated that the intestinal bacterial communities were essentially distinguishable between the midgut and midgut contents of *B. mori*, similar to *A. pernyi*, with a significantly greater richness and diversity in the midgut than in the midgut contents (the number of genera assigned for paired-sample *t* test; *t* = 4.258, *df* = 5, P = 0.008).

In *B. mori*, the boxplot of species richness and community diversity indicated a significant difference between midgut and midgut contents (Fig. 3a). PCoA analysis also indicated that the intestinal bacterial communities between the midgut and the midgut contents did indeed cluster separately (Fig. 3b). The nonparametric multivariate statistical tests (ANOSIM test with 999 permutations) further indicated a significant difference in the bacterial community between the midgut and midgut contents.

The dataset of *B. mori* revealed that the dominant bacterial phyla in the midgut and the midgut contents were Firmicutes (43.92% and 57.25%), Cyanobacteria (25.94% and 37.06%), Proteobacteria (21.39% and 3.88%), Actinobacteria (2.18% and 1.37%), and Bacteroidetes (0.87% and 0.23%). Among the five phyla, Proteobacteria and Bacteroidetes exhibited significant differences (P < 0.05) between sample types for *B. mori* (Fig. 4c). As shown in Fig. 4d, the dominant bacterial genera (abundance > 1%) in the midgut and the midgut contents were *Staphylococcus* (41.83% and 56.95%), *norank_c__Cyanobacteria* (27.28% and 37.08%), *Ralstonia* (8.51% and 1.74%), *Pseudomonas* (6.08% and 0.086%), *unclassified_k_norank* (4.63% and 0.01%), and *Burkholderia-Paraburkholderia* (1.22% and 0.19).

## Discussion

Here, we compared for the first time the diversity of insect intestinal bacterial communities between the midgut and the midgut contents. Insect intestinal bacterial communities have been attracting increasing interest with the advances in NGS. However, either the midgut or the midgut contents were used as material in previous studies^5,9,15,17^. The discrepancy in sampling material mainly depended on the author’s personal bias or laboratory tradition. At the beginning of this study, we were met with confusion on whether to choose the midgut or midgut contents. This confusion thus stimulated us to compare the intestinal bacterial communities between them. Our results from two silkworm species, *A. pernyi* and *B. mori*, indicated that the midgut possesses a (significantly for *B. mori*) greater bacterial composition and diversity than the midgut contents. We found that at the phylum level, almost all the bacterial compositions found in the midgut contents were well represented in the midgut in both silkworms, but eleven and nine phyla were midgut-specific for *A. pernyi* and *B. mori*, respectively. The majority of bacterial genera (77–85%) found in the midgut contents could also be represented in the midgut in both silkworms; 136 and 211 genera were midgut-specific, but only 10 and 90 genera were midgut content-specific for *A. pernyi* and *B. mori*, respectively. Therefore, we reached the conclusion that analysis of the midgut would be better to obtain a full view of the intestinal bacterial communities of insects in future research work.

This study provides the first information about the bacterial diversity associated with *A. pernyi* larvae reared in the field on oak leaves. The Chinses oak silkworm was closely associated with diverse populations of microbes and dominated by five bacterial phyla (Proteobacteria, Firmicutes, Cyanobacteria, Bacteroidetes, and Actinobacteria), as observed in *B. mori*^9^ and other insects^25,26^. These five phyla were also the most prevalent phyla in *B. mori* larvae reared in Shenyang, China, surveyed in this study. These results suggested that, at the level of the phylum, there were no dramatic changes in the midgut microbiota composition between the two silkworms. However, at the genus level, *B. mori* was dominated by *Staphylococcus* (42.34%), while *A. pernyi* was dominated by *norank_c__Cyanobacteria* (86.22%), also suggesting that the microbiota associations are influenced by a silkworm-specific effect. The number of shared OTUs between the midgut and midgut contents of the *B. mori* larvae samples was much higher than that of the *A. pernyi* samples, indicating that the diversity, composition and structure of bacterial communities in *B. mori* larvae might be higher than those in *A. pernyi* larvae*.*

The large size of the two silkworms provides a chance to use one single larva for sample preparation. Our results based on PCoA analysis also revealed highly similar intestinal bacterial communities among *B. mori* individuals but a discrete pattern among *A. pernyi* individuals. Microbiome acquisition occurs over evolutionary time and reflects complex feedbacks between the environment, diet, immune response, and strain^6–9^. A very low genetic diversity for the *B. mori* strain and a relatively high genetic diversity for the *A. pernyi* strain have been uncovered in a previous comparative study based on molecular marker analysis^27^. Our results might be explained by the purity of the present-day strain and the rearing conditions. The highly similar intestinal bacterial communities among *B. mori* individuals could be due to the genetically small difference within the strain and indoor-rearing style, and the discrete pattern among *A. pernyi* individuals could be due to the genetically high difference within the strain and outdoor-rearing style. Based on an increasing number of studies on the midgut microbiome in Lepidoptera, the evolutionary history of invertebrate-microbe symbioses will be revealed.

An interesting finding is that *Ralstonia* was one of the dominant bacteria in all surveyed samples. Bacteria in insects were originally derived from free-living environmental microorganisms^28^. *Ralstonia* bacteria, which are mostly related to the pathogenicity of plants, have a broad host range of over 50 families and cause serious harm worldwide^29^. Thus, we speculate that *Ralstonia* present in all samples surveyed in this study are derived from the leaf surface of the host plants, including *Morus alba* for *B. mori* and *Quercus wutaishanica* for *A. pernyi*.

To our knowledge, this is the first study to compare the diversity of insect intestinal bacterial communities between the midgut and midgut contents. Our data from *B. mori* larvae revealed that the midgut harbors a significantly greater bacterial composition and diversity than the midgut contents. More work should be performed to investigate whether similar trends would occur in other insects and explore the significance of the difference in the bacterial communities between the midgut and the midgut contents in the future.

## Materials and methods

### Experimental insects and rearing conditions

Two silkworm species, *A. pernyi* and *B. mori*, were used in this study. The *A. pernyi* inbred strain Yuda No. 1 was provided by the Henan Sericultural Research Institute, Zhengzhou, China, and completed two generations at the Department of Sericulture, Shenyang Agricultural University, Shenyang, China. The larvae of *A. pernyi* were reared on oak trees (*Q. wutaishanica*) in the field at the Silkworm Experimental Field of Shenyang Agricultural University (N41°50′3.00″; E123°34′18.03″). The *B. mori* inbred strain Dazao was provided by the Silkworm Gene Bank, Southwest University, Chongqing, China. The larvae of *B. mori* were reared on fresh mulberry leaves (*M. alba*) after hatching in a rearing room under normal conditions (25 °C, 70% humidity with a 14 h/10 h L/D cycle).

### Sample preparation

The fifth instar larvae were chosen in this study since they represent the mature larvae for the two silkworm species. The larvae were fed on the same host species since hatching. The fifth instar larvae of day 6 for *A. pernyi* and day 3 for *B. mori*, before the gluttonous stage, were dissected under aseptic conditions. In the first step, the midgut contents (including the peritrophic membrane, food particles, and digestive fluids) of each larva were directly placed into a 1.5 mL sterile centrifuge tube. Then, the remaining midgut (excluding the PM and including the midgut cells and the ectoperitrophic space) was washed with sterile saline three times and placed into a 1.5 mL centrifuge tube. Due to the large size of the two silkworms, we used one single larva for sample preparation. Immediately after collection, samples were stored at − 70 °C until use. All specimens were randomly selected, and the experimental sample was collected individually for further analysis.

### Total DNA extraction, PCR amplification and sequencing

Frozen samples packed in dry ice were transported to Majorbio BioTech Co., Ltd. (Shanghai, China) for DNA extraction, library preparation and DNA sequencing. Total DNA was extracted using a Z.E.N.A Soil DNA Kit (Omega Bio-tek, Norcross, GA, USA). The final DNA concentration and purification were determined by a NanoDrop 2000 UV–vis spectrophotometer (Thermo Scientific, Wilmington, USA), and the DNA quality was checked by 1% agarose gel electrophoresis. Based on the pretest, the amplified total DNA products were used as the template. PCR amplifications were conducted with the primer set 338F (5′-ACTCC TACGG GAGGC AGCAG-3′) and 806R (5′-GGACT ACHVG GGTWT CTAAT-3′), which targets the V3-V4 region of bacterial 16S rRNA and amplifies a product of 468 bp^30^^,^ and a thermocycler PCR system (ABI GeneAmp 9700, Life Technologies, USA). The PCRs were performed in a total volume of 20 μL containing 10 ng of template DNA, 4 μL of 5× FastPfu Buffer, 2 μL of 2.5 mM dNTPs, 0.8 μL of each primer (5 μM), and 0.4 μL of FastPfu Polymerase. The PCRs were conducted with the following procedure: 95 °C for 3 min, followed by 29 cycles at 95 °C for 30 s, 55 °C for 30 s, and 72 °C for 45 s and a final extension at 72 °C for 10 min. The amplified products were purified and quantified and homogenized using PicoGreen fluorometry. All steps contained the negative controls, and no contamination was observed. PCR product sequencing was accomplished using an Illumina MiSeq PE300 platform following the manufacturer's instructions.

### Data processing

The sequence data were analyzed on the Majorbio I-Sanger Cloud Platform (www.i-sanger.com), a free online platform. Raw fastq files were demultiplexed, quality-filtered by Trimmomatic and merged by FLASH (https://sourceforge.net/projects/flashpage/) with the following criteria: (i) The reads were truncated at any site receiving an average quality score < 20 over a 50 bp sliding window. (ii) Primers were exactly matched allowing 2 nucleotide mismatches, and the reads containing ambiguous bases were removed. (iii) Sequences whose overlap was longer than 10 bp were merged according to their overlap sequence. The 16S rRNA gene sequences obtained in this study have been deposited in the Sequence Read Archive (SRA) under the accession numbers SRR9082165-SRR9082169 for *A. pernyi* and SRR9095152-SRR9095163 for *B. mori*.

Operational taxonomic units (OTUs) were clustered with a 97% similarity cutoff using UPARSE (version 7.1 https://drive5.com/uparse/), and chimeric sequences were identified and removed using UCHIME^31^. The taxonomy of each 16S rRNA gene sequence was analyzed by the RDP Classifier algorithm (https://rdp.cme.msu.edu/) against the Silva (SSU123) 16S rRNA database using a confidence threshold of 70%^32^.

Alpha diversity is an important feature of bacterial community structure. It can reflect the abundance and diversity of bacterial flora through species diversity in a single sample. The Chao index, Ace index, Shannon index and Simpson index were used to analyze the richness and diversity of the intestinal bacteria^33–36^. The Chao index and the Ace index were commonly used to estimate the total number of species, and the Shannon and Simpson indices were used to estimate bacterial diversity in the sample. Simpson’s index negatively correlates with bacterial diversity.

## Supplementary information

Supplementary information
