# Supplementary material for: Comparison of bacterial communities between midgut and midgut contents in two silkworms, Antheraea pernyi and Bombyx mori
Source: Sci Rep. 2020 Jul 31;10:12966. doi: 10.1038/s41598-020-69906-y (PMC7395759; doi:10.1038/s41598-020-69906-y)
Supplement: Supplementary file 1 — Supplementary information [file 41598_2020_69906_MOESM1_ESM.pdf]

Supplementary information

Comparison of Bacterial Communities between Midgut and Midgut Contents in Two Silkworms, *Antheraea pernyi* and *Bombyx mori*

Huan Wang [†,1], Jing-Yu Zhang [†,1], Xiao-Meng Wang[†, 1], Hua-Lei Hu [1], Run-Xi Xia [1], Qun Li [1], Xu-Wei Zhu [2], Tian-Mao Wang [3], Yan-Qun Liu [*,1], Li Qin [1]

[1] Department of Sericulture, College of Bioscience and Biotechnology, Shenyang Agricultural University, 120 Dongling Road, Shenyang 110866, China

[2] Henan Sericultural Research Institute, Zhengzhou 450008， China

[3] Heilongjiang Sericultural Research Institute, Ha'erbin 150086, China

[†] These authors contributed equally to this work.

* Author for correspondence

Yan-Qun Liu, PhD. Department of Sericulture, College of Bioscience and Biotechnology, Shenyang Agricultural University, 120 Dongling Road, Shenyang 110866, China. Email: liuyanqun@syau.edu.cn.

Supplementary Tables

Supplementary Table 1. Composition of intestinal bacterial communities of silkworm larvae.

Supplementary Table 1.

Table 1. Composition of intestinal bacterial communities of silkworm larvae.

**Sample**		**Sequences**	**Phylum**	**Class**	**Order**	**Family**	**Genus**	**OTU**
A. pernyi midgut	_1	40,091	9	14	27	39	50	54
	_2	29,184	15	26	58	83	124	150
	_3	20,842	14	19	41	63	76	89
	Total	90,117	17	29	65	110	162	206
A. pernyi midgut contents	_2	29,959	5	9	14	16	19	22
	_3	32,651	6	9	17	23	23	26
	Total	62,610	7	11	24	31	36	42
B. mori midgut	_1	48,552	18	34	65	115	184	233
	_2	51,710	22	41	74	126	212	274
	_3	40,896	22	38	72	121	204	249
	_4	39,957	23	45	84	134	222	270
	_5	52,896	20	39	78	127	226	293
	_6	41,892	20	34	72	122	201	264
	Total	275,903	30	60	130	237	465	676
B. mori midgut contents	_1	48,412	12	22	45	77	119	143
	_2	55,549	10	20	41	64	88	102
	_3	71,184	14	25	50	90	156	191
	_4	52,082	15	31	71	132	205	268
	_5	43,694	11	20	48	88	137	162
	_6	48,331	17	27	55	97	153	187
	Total	319,852	22	43	95	180	344	481
